# The Effect of Chromium Picolinate Supplementation on the Pancreas and Macroangiopathy in Type II Diabetes Mellitus Rats

**DOI:** 10.1155/2014/717219

**Published:** 2014-06-25

**Authors:** Shan Huang, Wenfang Peng, Xiaohong Jiang, Kan Shao, Lili Xia, Yubin Tang, Jiayin Qiu

**Affiliations:** Shanghai Tong Ren Hospital Affiliated to Shanghai Jiaotong University Medical School, Xianxia Road No. 1111, Changning District, Shanghai 200336, China

## Abstract

*Purpose*. The aim was to explore the effect of the chromium picolinate (CrPic) administration on the pancreas and macroangiopathy of type II diabetes mellitus rats. *Methods*. The type II diabetes mellitus (T2DM) rat model was induced by low-dose streptozotocin (STZ). The rats were randomly divided into 5 groups (ten rats in each group). After supplementing CrPic for 15 weeks, the histopathological examination was performed by hematoxylin-eosin (HE) staining. Serum insulin and NO level were determined by radioimmunoassay and colorimetry, respectively. Serum glycosylated hemoglobin (HbA1C), adiponectin (APN), advanced glycation end products (AGES), and apelin were measured by ELISA. Real-time reverse transcription polymerase chain reaction (RT-PCR) was applied for detecting the mRNA expression of *APN* and *apelin*. *Results*. After CrPic treatment, compared with the T2DM control group (group 2), pancreas sections stained with HE showed the completed pancreatic cells structure and no inflammatory infiltration in groups 4 and 5. In addition, the levels of serum NO and insulin were significantly increased and the serum levels of HbA1C, AGES, APN, and apelin were significantly decreased in groups 4 and 5 compared with group 2. The mRNA expression of *APN* and *apelin* in groups 4 and 5 was also recovered to the normal level. *Conclusion*. CrPic can recover the function of *Β*-cells and alleviate macroangiopathy in STZ-induced T2DM rats.

## 1. Introduction

Diabetes mellitus (DM) is one kind of metabolic syndromes characterized by chronic hyperglycemia [1]. The cases of diabetes fall into two broad pathogenetic categories: type I and type II diabetes mellitus (T1DM and T2DM). T2DM, which is previously referred to as noninsulin-dependent diabetes mellitus (NIDDM), accounts for approximately 90% of the diabetes patients [2, 3]. There were many complications, such as nephropathy [4], cataracts [5], microangiopathy, and macroangiopathy [6] in T2DM. Among them, macroangiopathy is the most frequent complication in the patients with T2DM [7–9], which manifests as atherosclerosis like in nondiabetic patients and is characterized by formation of plaques that follows in stages but with an accelerated course due to the several risk factors [10].

In current clinical treatment, western medicines are widely used to control the hyperglycemia, hyperlipidemia, and insulin resistance of type 2 diabetes mellitus, such as sulfonylurea, biguanides, thiazolidinediones, and glycosidase inhibitors [11]. However, the clinical efficacy of these drugs in treating T2DM is limited. Thus, it is urgently needed to explore the novel drugs and therapies to improve the treatment effect and reduce the risk of complications of T2DM.

Chromium (Cr), as an essential element, is directly related to the activity of glucose tolerance factor (GTF) [12]. Cr can alleviate glucose intolerance and insulin resistance [13] and it is involved in the metabolism of glucose, lipid, protein, and nucleic acid [14–17]. However, as supplementary drug, Cr could not be effectively used due to the poor absorption rate (dietary chromium: 0.4–2%; chromium chloride: 0.5–2%) [18–20]. Chromium picolinate (CrPic), also named as picolinic acid chromium, is a convenient form of chromium that is used more efficiently than some other forms of chromium [10]. The absorption rate of it is about 0.7–5.2% [19]. Several studies have proved that CrPic, as the source of Cr, can alleviate the high level of blood glucose, blood lipid, insulin, and cholesterol in the patients with metabolic syndrome [10, 21, 22]. Therefore, the efficacy of CrPic in treating T2DM is undisputed. However, the efficacy of CrPic in treating macroangiopathy in patients with T2DM has not been known.

In the present study, we evaluated the effect of CrPic on pancreas and macroangiopathy in T2DM rat model by detecting the levels of serum and fat markers of T2DM. The model was induced by streptozotocin (STZ).

## 2. Materials and Methods

### 2.1. Animals and Diets

Male Wistar rats (*n* = 50) weighting 250 ± 20 g were purchased from the animal center of Chinese academy of science. The animals were reared in a specific pathogen-free (SPF) laminar flow cabinet with temperature of 25 ± 1°C, humidity of 40–60%, and a 12:12 h light-dark cycle. The conventional diet and sterilized water were used to feed these rats.

The conventional diet and the high fat diet (HFD) were purchased from the laboratory animal center of Peking Union Medical College (PUMC). The conventional diet contains 41.47% carbohydrate, 14.42% fat, and 21.06% protein. The HFD contains 10% lard, 20% sucrose, 1% Choline chloride, 2.5% cholesterol, and 66.5% conventional diet.

### 2.2. Model Establishment of T2DM, Grouping, and Sample Collection

After adaptive feeding for one week, the rats were divided into five groups of ten rats in each group as follows: group 1, normal control rats; group 2, T2DM control rats; group 3, T2DM rats supplemented with 25 *μ*g/kg CrPic; group 4, T2DM rats supplemented with 50 *μ*g/kg CrPic; and group 5, T2DM rats supplemented with 100 *μ*g/kg CrPic. Ten rats of normal control group were fed with conventional diet. Forty rats of the other four groups were fed with HFD. After four weeks, the forty rats of the other four groups were injected intraperitoneally with low dose (30 mg/kg) of STZ (Sigma) which was prepared in sterile citrate buffer (w/v: 2%). Ten rats of the normal control group were injected intraperitoneally with the same volume of sterile citrate buffer. After 72 h, the peripheral blood glucose level in tails of rats was detected by the Precision Xtra blood glucose monitoring system (Alameda, CA, USA). The model was successfully established if the blood glucose of the rats was over 16.7 mmol/L and polyuria and polydipsia appeared in these rats. After the levels of blood glucose stably were maintained for one week, the blood glucose and serum insulin concentrations of the rats in each group were determined before supplementing CrPic (Spectrum chemical & laboratory products, China). The rats of groups 3, 4, and 5 were supplemented with CrPic by gavage with equal volume of normal saline (NS; 0.9% NaCl) that contained different concentration of CrPic at every morning for 15 weeks. The rats of groups 1 and 2 were only supplemented with equal volume of NC that did not contain CrPic. After last time of CrPic administration, the rats were fasted but were not prohibited from drinking water for 12 h. The weight change and food intake of the rats in each group were monitored once a week and the behaviors and mental state of all the rats were observed during the whole experiment. Finally, all the rats were decapitated and the blood and the fat from the abdomen were collected for biochemical analyses. The pancreas was collected for hematoxylin-eosin (HE) staining.

### 2.3. Histopathological Analysis

The histopathological examinations of the pancreatic sections of the rats in each group were performed by standard histological techniques with HE staining. The collected pancreas tissue was fixed in 10% buffered formalin and embedded in paraffin and sections of pancreatic tissue were deparaffinized and stained with HE. The pathological changes of the lesion and its vicinity were observed by the light microscopy.

### 2.4. Determination of Serum Markers of T2DM

The detection of serum nitric oxide (NO) level was detected by using the Nitric Oxide Colorimetric Assay Kit (Nanjing Jiancheng Bioengineering Institute, China) following manufacturer's instruction. The serum insulin level was determined by using the radioimmunoassay kit (China Institute of Atomic Energy) according to the procedure described by the manufacturer. The serum levels of glycosylated hemoglobin (HbA1C), advanced glycation end products (AGES), adiponectin (APN), and apelin were assayed with an ELISA kit (R&D System, Minneapolis, MN).

### 2.5. Determination of mRNA Expression of APN and Apelin

The fat tissues obtained from abdomen were processed for RNA extraction. The TRIzol reagent (Invitrogen, Carlsbad, CA) was used to extract total RNAs for analyzing the mRNA expression of APN and apelin mRNA. The cDNA was synthesized out of the total RNA with a cDNA synthesis kit (Promega, Southampton, UK). The  *β* *-actin* was regarded as control. The primer sequences of* APN*, apelin, and  *β* *-actin* were shown in Table 1. Real-time reverse transcription polymerase chain reaction (RT-PCR) was performed using a reverse transcription Kit (Promega, Southampton, UK) following the manufacturer's protocol. The reaction condition of the real-time RT-PCR was 40 cycles of 95°C for 15 s, 55°C for 15 s, and 72°C for 15 s. Relative quantification of gene expression was done using the comparative CT (2^−ΔΔCT^) method.

### 2.6. Statistical Analyses

The data was analyzed by using the SPSS 13.0 software. Comparisons among four experimental groups were analyzed by one-way analysis of variance (ANOVA) followed by Bonferroni's test to evaluate statistical difference between two groups. *P* values less than 0.05 were defined as statistically significant. All data are presented as mean ± standard deviation.

## 3. Results

### 3.1. Establishment of T2DM Rat Model

After injecting STZ for 72 h, the average concentrations of blood glucose of the rats in groups 2 to 5 all reached 16.7 mmol/L. Then, the levels of blood glucose were stably maintained for one week. The blood glucose and serum insulin concentrations of the rats are shown in Table 2. Compared with group 1, the blood glucose and serum insulin concentrations of the rats in the other four groups were significantly higher, which indicated that the rats suffered T2DM induced by STZ. In addition, the apathetic state and behaviors of polyuria and polydipsia appeared in the rats of groups 2 to 5. Therefore, the T2DM rat model was successfully established.

### 3.2. Effects of Chromium Picolinate on Histopathology of Pancreas

**Figure 1:**
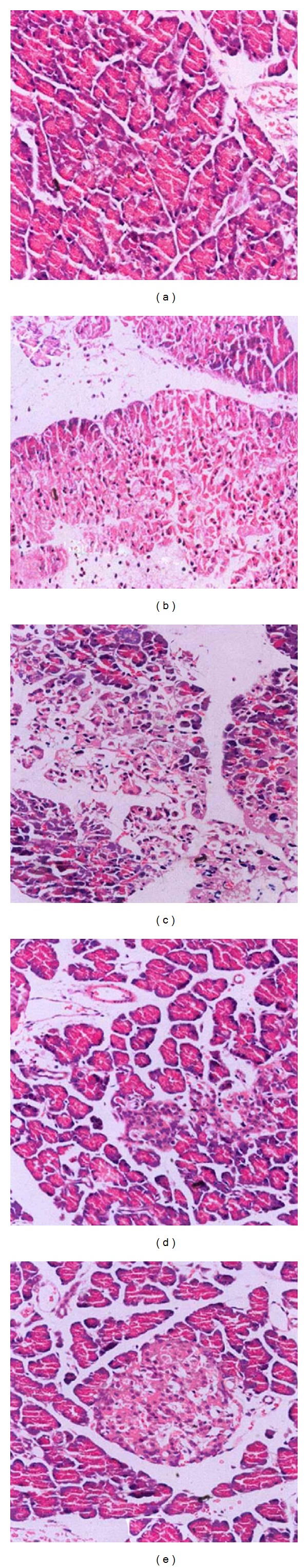
The HE staining of pancreas (HE 400x) in five groups. (a) Group 1, normal control group; (b) group 2, T2DM (type II diabetes mellitus) control group; (c) group 3, 25 *μ*g/kg CrPic group; (d) group 4, 50 *μ*g/kg CrPic group; and (e) group 5, 100 *μ*g/kg CrPic group.

The pancreatic sections stained with HE are shown in Figure 1. It showed that the rats in group 1 had normal pancreatic endocrine and exocrine architecture, acinar cells, and pancreatic islets. Moreover, there was no edema or inflammatory cells infiltration (Figure 1(a)). However, administration of STZ caused necrotic changes (karyolysis and disappearance of nucleus) of pancreatic islets, degeneration of acinar cells, and inflammatory cells infiltration in STZ-induced T2DM rats (Figure 1(b): group 2). After CrPic treatment, some improvements on the morphology of pancreas were observed. In the rats supplemented with 50 *μ*g/kg (Figure 1(d): group 4) and 100 *μ*g/kg CrPic (Figure 1(e): group 5), there was completed pancreatic cells structure and no inflammatory cell infiltration was observed. In the rats supplemented with 25 *μ*g/kg CrPic (Figure 1(c): group 3), the improvement on the morphology of pancreas was not significant compared with the rats supplemented with 50 *μ*g/kg and 100 *μ*g/kg CrPic.

### 3.3. Effects of Chromium Picolinate on Serum Markers

**Figure 2:**
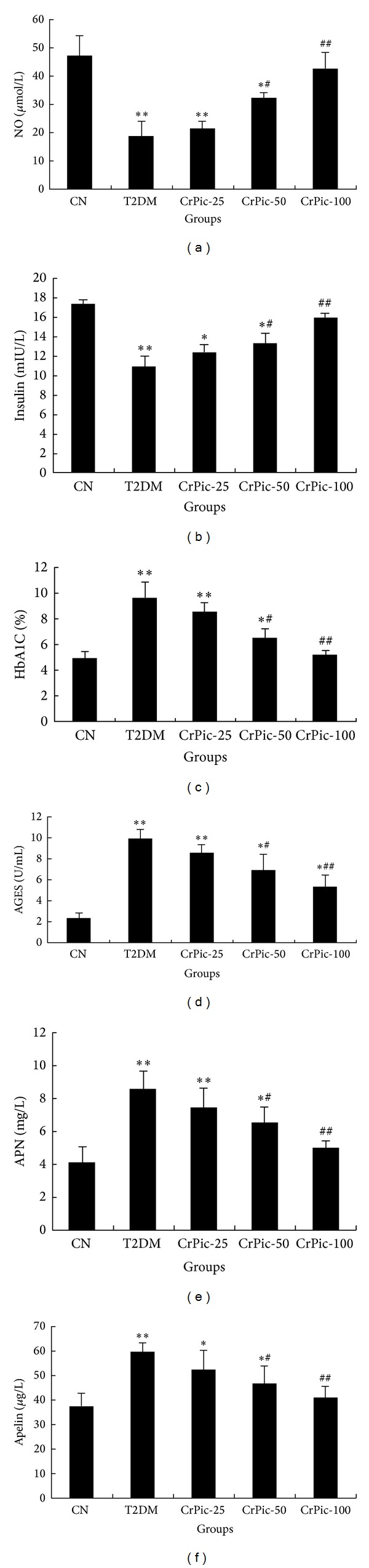
The effect of CrPic on the levels of serum NO (a), insulin (b), HbA1C (c), AGES (d), APN (e), and apelin (f). CN: the normal control group, group 1; T2DM: T2DM (type II diabetes mellitus) control group, group 2; CrPic-25: group 3 (25 *μ*g/kg); CrPic-50: group 4 (50 *μ*g/kg); and CrPic-100: group 5 (100 *μ*g/kg). ^∗∗^ *P* < 0.01 compared with group 1; ^∗^ *P* < 0.05 compared with group 1; ^##^ *P* < 0.01 compared with group 2; and ^#^ *P* < 0.05 compared with group 2.

Compared with group 1, NO and insulin level of the rats in group 2 were significantly decreased (*P* < 0.01). After CrPic treatment, the NO and insulin levels of the rats in groups 4 (*P* < 0.05) and 5 (*P* < 0.01) were significantly increased compared with group 2. The NO and insulin level of the rats in group 3 were also higher than those in group 2, but the statistical difference was not significant. The dose dependent effect was observed in the effect of CrPic on NO and insulin level of STZ-induced T2DM rats which were raised with the increase of the dose of CrPic. The NO and insulin level of the rats in group 5 were very close to group 1 (Figures 2(a) and 2(b)).

According to Figures 2(c), 2(d), 2(e), and 2(f), the levels of HbA1C, AGES, APN, and apelin of rats in group 2 were significantly higher than those in group 1 (*P* < 0.01). After CrPic treatment, the levels of HbA1C, AGES, APN, and apelin of the rats in groups 4 (*P* < 0.05) and 5 (*P* < 0.01) were significantly reduced compared with group 2. The dose dependent effect was also observed in the effect of CrPic on levels of HbA1C, AGES, APN, and apelin of STZ-induced T2DM rats which were reduced with the decrease of the dose of CrPic. The decrease of the levels of HbA1C, AGES, APN, and apelin of the rats in group 3 was not significant compared with group 2. The levels of HbA1C, AGES, APN, and apelin level of the rats in group 5 were almost close to group 1.

### 3.4. Effects of Chromium Picolinate on the mRNA Expression of APN and Apelin

**Figure 3:**
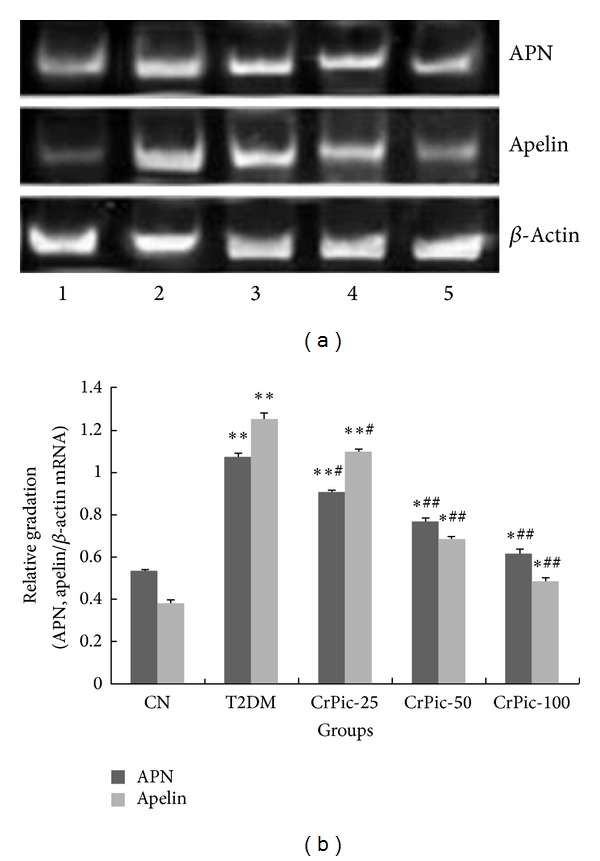
The mRNA expression levels of APN and apelin in each group. (a) Agarose gel electrophoresis of RT-PCR products. (b) The histogram of relative mRNA expression levels of APN and apelin compared with *β* *-actin*. CN: the normal control group, group 1; T2DM: T2DM (type II diabetes mellitus) control group, group 2; CrPic-25: group 3 (25 *μ*g/kg); CrPic-50: group 4 (50 *μ*g/kg); and CrPic-100: group 5 (100 *μ*g/kg). ^∗∗^ *P* < 0.01 compared with group 1; ^∗^ *P* < 0.05 compared with group 1; ^##^ *P* < 0.01 compared with group 2; and ^#^ *P* < 0.05 compared with group 2.

The mRNA expression levels of* APN* and apelin were significantly higher in group 2 than those in group 1 (*P* < 0.05). After CrPic treatment, the mRNA expression levels of* APN* and apelin of the rats in groups 3 (*P* < 0.05), 4 (*P* < 0.01), and 5 (*P* < 0.01) were significantly decreased compared with group 2 (Figure 3).

### 3.5. The Weight Change and Food Intake of the Rats

The weight change and food intake of the rats in each group during the whole experiment are shown in Table 3. Compared with group 1, the weight changes of the rats in the other four groups were significantly less while the food intake was significantly more. After the administration of CrPic, the weight changes and food intake of the STZ-induced T2DM rats in groups 3 to 5 were gradually close to group 1 with the increase of the dose of CrPic.

## 4. Discussion

T2DM is a metabolism syndrome with multifactors and involved multiorgans, which is characterized by chronic high blood glucose. Macroangiopathy was usually seen as the complication in the patients with diabetes. The macroangiopathy is one of the main reasons of death and disability [23, 24]. In addition, the pathogenesis of T2DM was very complicated and associated with insulin resistance and secretion deficiency [25, 26], inflammatory reaction [27], and oxidative stress [28]. Therefore, it is critical to find the suitable therapy of treating T2DM and macroangiopathy based on the pathogenesis of T2DM.

CrPic is organic chromium compound with small molecular weight and low toxicity. It is promising in therapy application because it can increase the activity of insulin and slow down the development of diabetes mellitus [29]. In the present study, the T2DM model was induced by STZ; STZ liberates toxic amounts of nitric oxide that inhibits aconitase activity and participates in DNA damage [30]. As a result of the STZ action, *β*-cells undergo destruction by necrosis. After CrPic supplement, the inflammatory cells infiltration was reduced or appeared and the function of *β*-cells was recovered.

For the levels of serum markers of T2DM, the results provided evidence for the ability of CrPic in regulating the levels of NO, insulin, HbA1C, AGES, APN, and apelin. T2DM frequently results from progressive failure of pancreatic *β*-cell function in the presence of chronic insulin resistance [31]. Some previous studies have reported that CrPic supplementation could increase insulin sensitivity in subjects with type 2 diabetes [22, 32, 33]. The secretion of insulin comes from *β*-cell. Therefore, we speculated that the level of insulin was raised after CrPic treatment due to the increase of insulin sensitivity in T2DM rats and the improvement of *β*-cell function. In addition, HbA1C, as another indicator of diabetes, is the product of hemoglobin and glucose in the red blood cells and it can be used as the reference standard of average blood glucose level. Previous study showed that 1% decrease of the HbA1C resulted in 21% decrease in diabetes related end-point event, and the risk of the diabetes related death and myocardial infarct will decrease 21% and 14%, respectively [34]. After reconfiguration, the HbA1C can transform into AGES, so it is positively related to AGES. Then AGES can interact with its receptor and multiple cytokines and growth factors will be produced. Some articles reported that AGES could lead to vascular hyperplasia and accelerate the atherosclerosis [35, 36]. Furthermore, the increase of AGES level can result in decreasing level of NO. Serum NO is one of the inflammatory cytokines. Some clinical and epidemiological studies suggest that some processes related to low-grade inflammation may be relevant to diabetic micro- and macroangiopathy [37, 38]. Thus, the macroangiopathy was associated with the levels of serum NO. In this study, the decrease of NO level in STZ-induced T2DM rats might cause the change of angiotasis and then the macroangiopathy happened. The efficacy of CrPic on treating macroangiopathy was proved by the increase of serum NO level after CrPic treatment. Moreover, the changes of these markers were all dose dependent. In summary, CrPic may affect macroangiopathy in T2DM by increasing the NO level and decreasing the HbA1C and AGES level.

APN and apelin are the cytokines secreted by adipocytes. It was reported that the increase of the APN was observed in the patients with coronary artery disease and early atherosclerosis [39, 40]. In this study, the STZ injection with HFD will induce the mRNA expression of* APN* and apelin; it indicated that the macroangiopathy might occur with the T2DM. With the treatment of CrPic, the expression of the two genes was recovered back to normal level. These results indicated that the effect of CrPic on macroangiopathy in T2DM may be associated with the regulation of the lipid metabolism.

Furthermore, the weight change and food intake were also improved after CrPic treatment. The weight gain of rats was attenuated by STZ administration. It may be caused by the decrease of insulin. The dysregulation of insulin on blood glucose may affect the conversion of glucose and lipid metabolism [41]. Thus, the weight gain was decreased after STZ administration. Then, the weight gain was recovered to normal with the CrPic treatment. Based on the results of this study, the food intake was decreased after CrPic supplement. Thus, we inferred that the mechanism of the changes of weight gain in T2DM rats might be related to the effect of CrPic on the serum and fat markers such as NO, insulin, HbA1C, AGES, APN, and apelin.

## 5. Conclusion

In conclusion, CrPic can recover the function of *β*-cells and alleviate macroangiopathy in STZ-induced T2DM rats. Further studies were required to verify the results of this study.

## Figures and Tables

**Table 1:** The primer sequences of the APN, apelin, and *β*-actin.

Gene name	Forward primer	Reverse primer
*APN *	CTG GAG AGA AGG GAG AGA AG	GCT GAA TGG TGA GTG ATA CA
*Apelin *	CTG CTC TGG CTC TCC TTG AC	ATG GGT CCC TTA TGG GAG AG
*β* *-Actin *	TCT TCC AGC CTT CCT TCC TG	TAG AGC CAC CAA TCC ACA CA

APN: adiponectin.

**Table 2:** The blood glucose and serum insulin concentrations of the rats after the blood glucose levels were stably maintained for one week.

Group	CN	T2DM	CrPic groups		
			(25 *μ*g/kg)	(50 *μ*g/kg)	(100 *μ*g/kg)
Blood glucose (mmol/L)	4.63 ± 1.02	16.81 ± 3.65∗	17.52 ± 2.76∗	17.91 ± 2.89∗	18.03 ± 1.15∗
Serum insulin (mIU/L)	16.88 ± 0.83	11.51 ± 1.07∗	10.75 ± 1.23∗	9.98 ± 1.03∗	11.96 ± 0.74∗

CN: the normal control group, group 1; T2DM: T2DM (type II diabetes mellitus) control group, group 2; CrPic groups: group 3 (25 *μ*g/kg), group 4 (50 *μ*g/kg), and group 5 (100 *μ*g/kg); and *compared with group 1, *P* < 0.01.

**Table 3:** The weight change and food intake of the rats in each group during the whole experiment.

Group	CN	T2DM	CrPic groups		
			(25 *μ*g/kg)	(50 *μ*g/kg)	(100 *μ*g/kg)
Weight change (g/week)	397.80 ± 21.74	349.551 ± 13.25∗	357.32 ± 12.93∗	357.11 ± 14.50∗	360.03 ± 21.01∗
Food intake (g/day)	16 ± 3	42 ± 7∗	37 ± 5∗	29 ± 4∗	27 ± 7∗

CN: the normal control group, group 1; T2DM: T2DM (type II diabetes mellitus) control group, group 2; CrPic groups: group 3 (25 *μ*g/kg), group 4 (50 *μ*g/kg), and group 5 (100 *μ*g/kg); and *compared with group 1, *P* < 0.01.
